# Endothelial Dysfunction in Hypertension: Current Concepts and Clinical Implications

**DOI:** 10.3389/fmed.2021.798958

**Published:** 2022-01-20

**Authors:** Giovanna Gallo, Massimo Volpe, Carmine Savoia

**Affiliations:** Clinical and Molecular Medicine Department, Cardiology Unit, Sant'Andrea Hospital, Sapienza University of Rome, Rome, Italy

**Keywords:** endothelium, inflammation, angiotensin II, vascular function, reactive oxygen species (ROS)

## Abstract

Endothelium plays a fundamental role in the cardiovascular system, forming an interface between blood and adjacent tissues by regulating the vascular tone through the synthesis of nitric oxide, prostaglandins and other relaxing factors. Endothelial dysfunction is characterized by vasoconstriction, cell proliferation and shifting toward a proinflammatory and prothrombic state. In hypertension endothelial dysfunction may be involved in the initiation and development of vascular inflammation, vascular remodeling, and atherosclerosis and is independently associated with increased cardiovascular risk. Different conditions such as impaired vascular shear stress, inflammation and oxidative stress, activation of the renin angiotensin system have been described as important pathophysiological mechanisms involved in the development of endothelial dysfunction. The release of extracellular vesicles by neighboring cells in the vascular wall has emerged as an important regulator of endothelial function and with potential antihypertensive properties and beneficial effects by counteracting the hypertension mediated organ damage. Furthermore, macrovesicles are emerging as an innovative therapeutic approach for vascular protection, allowing the delivery of bioactive molecules, such as miRNA and drugs interacting with the renin angiotensin system. In this review we summarize the available evidence about the pathophysiological implications of endothelial dysfunction in cardiovascular diseases, focusing on hypertension and its sequelae, and the potential innovative therapeutic strategies targeting the endothelium with the aim to improve vascular function and remodeling.

## Introduction

Vascular endothelium plays an important role in cardiovascular (CV) physiology, forming an interface between blood and adjacent tissues and it is involved in nutrients and metabolites transport as well as in the interaction with circulating cells, hormones, and cytokines (1). Endothelial cells regulate the vascular tone through the synthesis of nitric oxide (NO), prostaglandins and other relaxing factors. Moreover, healthy endothelium provides antioxidant, anti-inflammatory, and antithrombotic functions and contributes to the maintenance of vascular tone, serving as a gatekeeper for organ/tissue homeostasis and blood pressure control (2).

Endothelial dysfunction is characterized by a shift of the actions of the endothelium toward reduced vasodilation, cell proliferation, platelet adhesion and activation and proinflammatory and prothrombic state. Endothelial dysfunction occurs in association with several CV risk factors, including hypertension, hypercholesterolemia and insulin resistance, contributing to inflammation in the vascular wall, of resistance arteries as well as to increased lipoprotein oxidation, smooth muscle cell proliferation, extracellular matrix deposition, cell adhesion, and thrombus formation in conducting arteries (3–5).

It should be noted that the manifestations of endothelial dysfunction may precede the development of hypertension (6). Essential hypertension is characterized by functional and structural alterations in resistance arteries which lead to increased peripheral vascular resistance (7). Endothelial dysfunction may contribute to the increased peripheral resistance by several mechanisms that leads to the enhanced constriction and vascular remodeling (i.e., structural, mechanical, and functional alterations) of resistance arteries, which is associated to the development and complications of hypertension (6, 8). In particular, endothelial dysfunction may participate to the increased myogenic tone of resistance arteries through the activation of the renin-angiotensin system (RAS), endothelin-1, catecholamines, and growth factors production, leading to vasoconstriction, vascular remodeling and then to increased resistance to blood flow and ultimately to increased peripheral blood pressure. The induction of inflammatory processes in the vascular wall may be associated to endothelial dysfunction and may contribute further to the remodeling of resistance arteries (6, 9), and conduit arteries which is associated with the increased risk of atherosclerosis and the development of CV disease (CVD) (6, 10–12).

In this review we will discuss the available evidence on the pathophysiological implications of endothelial dysfunction in hypertension, as well as the potential innovative therapeutic strategies targeting the endothelium.

## Mechanisms of Endothelial Dysfunction in Hypertension, and Therapeutic Intervention

### Mechanical Stimuli

**Figure 1:**
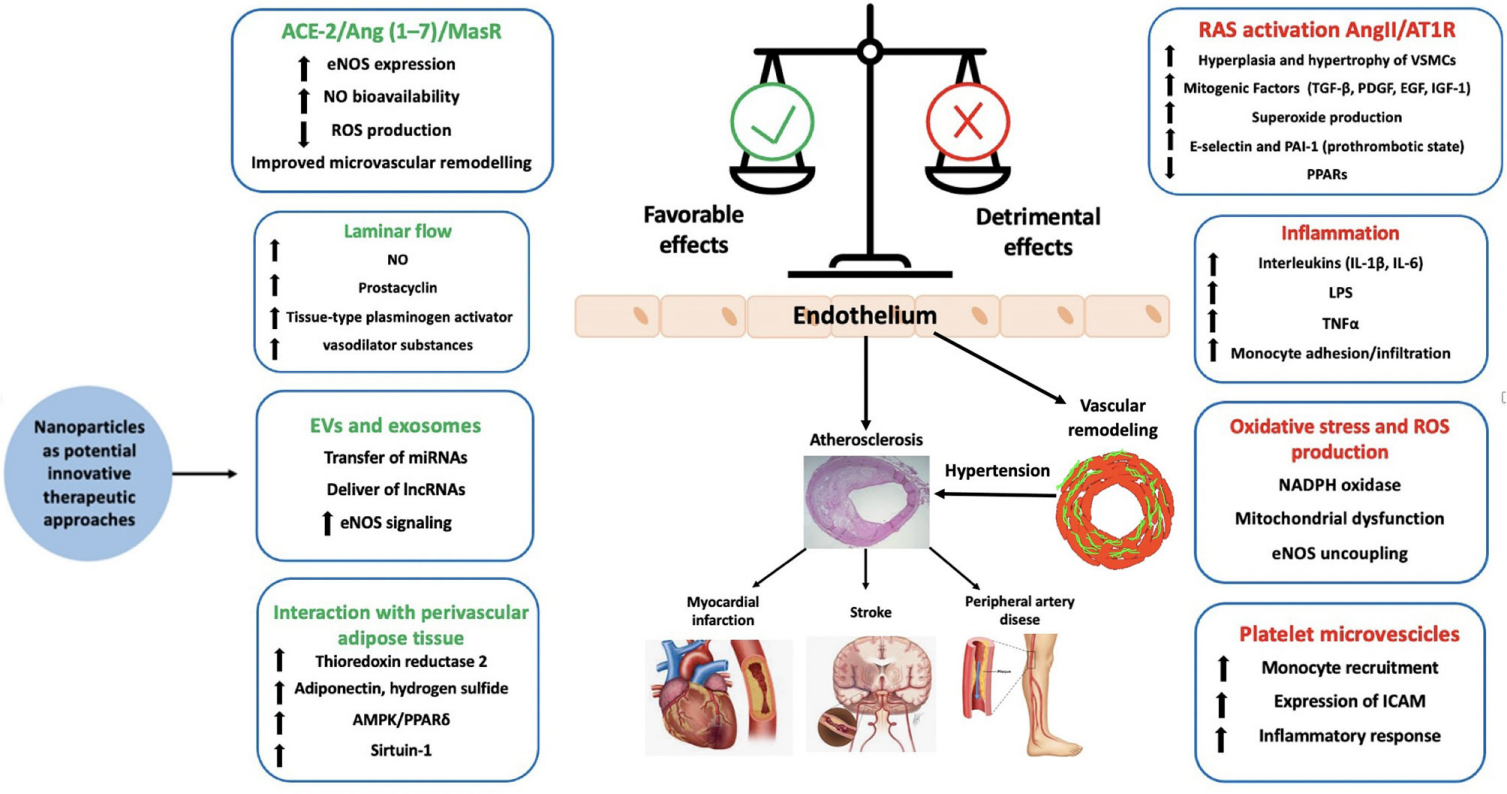
Factor contributing to endothelial function and dysfunction. ACE-2, angiotensin converting enzyme-2; AMPK, AMP-activated protein kinase; Ang, angiotensin; AT1R, type 1 angiotensin II receptor; EGF, epidermal growth factor eNOS, endothelial nitric oxide synthase; EVs, extracellular vesicles; ICAM, intercellular adhesion molecule 1; IGF-1, insulin-like growth factor-1; lncRNA, long-non-coding RNA; LPS, lipopolysaccharide; MCP-1, monocyte chemotactic protein 1; NAPDH, nicotinamide adenine dinucleotide phosphate; NO, nitric oxide; PAI-1, plasminogen activator inhibitor-1; PDGF, platelet derived growth factor; PPAR, peroxisome proliferator receptor; ROS, reactive oxygen species; TGF-β, tumor growth factor-β; TNFα, tumor necrosis factor.

Endothelial function is tightly regulated by the activation of several mediators and systems including NO, prostaglandins and other relaxing factors as well as by mechanical stimuli including vascular shear that stimulates numerous downstream signaling pathways to maintain and regulate endothelial function and vascular tone (13, 14). A fundamental distinction should be made between steady laminar and oscillatory flow (Figure 1). It has been shown that laminar flow enhances the production of vasodilator factors such as NO, prostacyclin, tissue-type plasminogen activator by the activation of mechanosensors and mechanosensitive channels which have been proposed to regulate a broad range of endothelial and vascular functions (15). Laminar shear activates the glycocalyx mechanosensing which is transferred by the cytoskeleton to integrins that distribute the force via actin microfilaments, microtubules, and intermediate filaments through the focal adhesion of c-Src kinases (15) leading to the maintenance of endothelial integrity. Increased laminar shear stress results also in elevated concentration of endothelial cytosolic calcium, leading to the activation of NO synthase (eNOS) and the increased production of NO. Elevated cytosolic calcium levels also trigger the opening of calcium-activated potassium channels, which is associated to endothelial cells hyperpolarization and thereby to vasorelaxation. Moreover, platelet endothelial cell adhesion molecule-1 (PECAM-1) along with caveolin, tyrosine-specific phospho-transferase Fyn, vascular endothelial growth factor (VEGF)-receptor 2 and the vascular endothelial cadherin (VE-cadherin) forms a mechanosensory complex which confers an adequate responsiveness to the beneficial effects of shear stress in endothelial cells (16). In particular, in laminar flow the force exerted on PECAM-1 triggers the activation of VEGF- receptor 2 in the absence of its ligand, which in turn induces integrin-mediated signaling and ultimately leads to the suppression of inflammatory pathways (17).

In response to increased shear stress, AMPK-induced phosphorylation and sirtuin-1-mediated deacetylation promoted eNOS compartmentalization and activation with atheroprotective effects in an *in vivo* mouse model (18), thus contributing to vascular protection. Moreover, laminar flow increases JNK-mediated p53 phosphorylation, GADD45 and p21cip1, inhibiting endothelial cells growth and atherosclerotic plaque development (19, 20).

On the other hand, oscillatory flow reduces eNOS expression, promotes leukocyte infiltration, smooth muscle proliferation and the secretion of proinflammatory molecules, such as MCP-1 (monocyte chemotactic protein 1), PDGFs (platelet derived growth factor), and endothelin-1 leading to vasoconstriction, increased blood pressure (BP) and atherosclerosis development in larger arteries (21). These processes involve the activation of mechanosensitive genes in endothelial cells, inducing the increase of reactive oxygen species (ROS) and the activation of several transcription factors, such as Kruppel-like factor [KLF2/4], NF-κB, AP-1, early growth response-1, c-Jun, c-fos, and c-myc, as well as the activation of mitogen-activated protein kinases (MAPKs) and small ubiquitin-like modifier (SUMO) signaling (15, 22–24). Interestingly the SUMOylation process can downregulate the expression of the protective transcription factor p53 which in turn can be associated to the development of CV complications in hypertension. Furthermore, oscillatory flow induces the activation of the PI3Kinase-Akt pathway which leads to the assembly of the nicotinamide adenine dinucleotide phosphate (NADPH) oxidase-2 and to the production of ROS (15), thus contributing to the vascular inflammation and remodeling. Other possible mechanisms involved in endothelial dysfunction triggered by oscillatory flow include the expression of the transcriptional factor Yes-associated protein (YAP) and its related coactivator PDZ binding motif (TAZ) that enhances cell cycle regulatory genes such as cyclin A1 (CNNA1) and E2F transcription factor 1 (E2F1) and increases inflammation and monocyte attachment, contributing in turn to endothelial dysfunction (15).

### Role of Oxidative Stress and Inflammation

A large body of evidence over the past years has shown that ROS are involved in endothelium dysregulation. In the vascular system the major source of ROS production is NADPH oxidase whose expression is increased in hypertensive conditions by several stimuli including shear stress alterations, renin angiotensin system (RAS) and endothelin activation (25).

ROS are key signaling molecules through which vasoactive agents such as angiotensin II (Ang II), endothelin-1 and prostanoids mediate effects at cellular level, and may modify cell function through highly regulated redox-sensitive signal transduction. This may occur through the alteration of intracellular calcium homeostasis contributing to vasoconstriction, cell growth and inflammation which lead to hypertension development and hypertension mediated organ damage (HMOD) (26, 27). ROS stimulate multiple signaling pathways involved in inflammation, cell growth and vascular remodeling. These pathways include the activation of NF-κB, MAPK, JAK-2, STAT, p21Ras, Pyk-2 (Proline-rich Tyrosine Kinase 2) and AKT, receptor tyrosine kinases such as EGFR (Epidermal Growth Factor Receptor), IGFR (Insulin-like Growth Factor Receptor 1) and PDGFR (Platelet Derived Growth Factor Receptor), protein tyrosine phosphatases and redox-sensitive transcriptor factors such as Activator Protein 1 (AP)-1 and Hypoxia-inducible factor 1 (HIF-1) (15, 22–24, 28–33).

In hypertension, oxidative stress promotes aberrant cell signaling and post-translational modification (oxidation and phosphorylation) of proteins and in turn cell and tissue damage (34). In particular, protein phosphatases such as tyrosine phosphatases and protein serine/threonine phosphatases are inactive in the oxidized state, resulting in increased phosphorylation and activation of downstream protein targets involved in cell growth and inflammation which may contribute to vascular remodeling and hypertension development (34, 35) (Figure 1). ROS can also inhibit SIRT1 activity through oxidative modifications on its cysteine residues. Decreased activity of SIRT1 enhances the NF-κB signaling, which supports inflammatory responses (36). Moreover, reduced SIRT1 activity is associated with a decreased AMP-activated protein kinase (AMPK) activation, which results in a reduced expression of antioxidant enzymes such as manganese superoxide dismutase, catalase, γ glutamylcysteine synthase, and thioredoxin (37).

Increased ROS concentration induces the reduction of NO bioavailability by the increased quenching (34). Furthermore, the ROS-dependent phosphorylation of ERK5 by phosphokinase-C-ζ (PKCζ) and the activation of tumor necrosis factor α (TNFα)- mediated pathway induces the degradation of eNOS leading to the reduced production of NO concentration and in turn contributing further to endothelial dysfunction (34).

### Role of Renin Angiotensin System and Its Antagonism

RAS and in particular its key effector Ang II play a fundamental role in the development of hypertension and its sequelae, contributing to endothelial dysfunction, cell growth, oxidative stress, vasoconstriction and inflammation. Ang II induces hyperplasia and hypertrophy of vascular smooth muscle cells (VSMC) in resistance arteries by modulating the endogenous production of mitogenic factors (including TGF-β (tumor growth factor-β), PDGF (platelet-derived growth factor), EGF (epidermal growth factor), IGF-1 (insulin-like growth factor 1) (38) and by enhancing basal superoxide production through the activation of cSrc, PKC, phospholipase A2 (PLA2) and phospholipase D (PLD) and increased NADPH oxidase and ROS generation (27, 39, 40). Moreover, Ang II stimulates the production of E-selectin and plasminogen activator inhibitor-1 (PAI-1), contributing to a prothrombotic state and to atherosclerotic plaque rupture (41). In addition, Ang II downregulates PPARs which have been largely demonstrated to reduce inflammatory response in experimental animals and to decrease serum markers of inflammation in humans (42). Through the stimulation of AT1 (Ang II type 1) receptors, Ang II also induces the synthesis of aldosterone which activates mineralocorticoid receptors enhancing inflammation, fibrosis, and endothelial damage (43).

As a matter of fact, RAS inhibitors and mineralocorticoid receptor antagonists have been demonstrated to reduce the proinflammatory and pro-fibrotic effects of Ang II and aldosterone, improving endothelial function and reducing oxidative stress (44).

Available evidence suggests that RAS blockade obtained by angiotensin converting enzyme (ACE) inhibitors or angiotensin receptor blockers (ARBs) is associated with improved function and structure of resistance arteries (6, 45, 46). The activation of complementary protective axes of the RAS may potentially contribute to the beneficial effects of RAS blockers. This includes the expression of angiotensin II type 2 receptor (AT2R) through the activation of a functional crosstalk between AT1R/AT2R during selective AT1R blockade (47–50). AT2R may contribute to improve endothelial dysfunction and arterial remodeling in hypertensive conditions, as its activation is linked to vasodilation, NO production and antiproliferative and anti-inflammatory effects (51). Thus, AT2R may participate to the mechanisms whereby therapeutic use of ARBs induces cardiovascular protection (52).

Experimental studies suggest that also the activation of ACE-2/Ang (1–7)/MasR axis may in part counteract the Ang II–induced actions in the cardiovascular system, including endothelial dysfunction, vasoconstriction and cell growth (53). In this regard, we have recently shown that ACE-2/Ang (1–7)/MasR axis plays an important role in arterial protection during selective AT1R blockade through the improvement of endothelial function and remodeling of resistance arteries *via* the reduction of ROS production and increased NO bioavailability (54) (Figure 1).

### Effect of Other Antihypertensive Drugs and Endothelial Dysfunction

**Figure 2:**
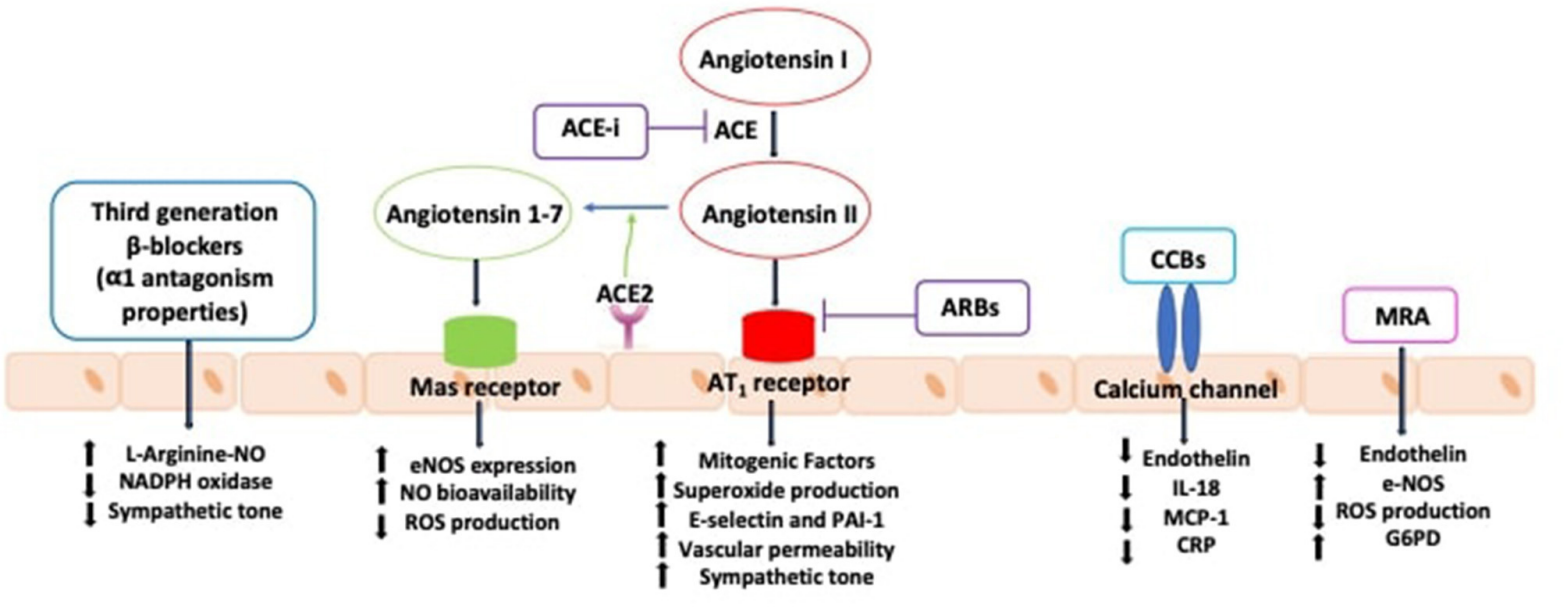
Effects of antihypertensive agents in improving endothelial function. ACE, angiotensin converting enzyme; ACE-i, angiotensin converting enzyme inhibitors; ARBs: angiotensin receptor blockers; AT1R, type 1 angiotensin II receptor; CCBs, calcium channel blockers; CRP, C-reactive protein; eNOS, nitric oxide synthase; G6PD, glucose-6-phosphate dehydrogenase; IL-18, interleukin-18; MCP-1, monocyte chemotactic protein 1; MRA, mineralocorticoid receptor antagonists; NAPDH, nicotinamide adenine dinucleotide phosphate; NO, nitric oxide; PAI-1, plasminogen activator inhibitor-1; ROS, reactive oxygen species.

Other antihypertensive agents recommended in clinical practice have also shown vascular protective effects. Mineralocorticoid receptor antagonists have been demonstrated to reduce arterial stiffness and to improve endothelial function, measured by flow-mediated dilation (55). Calcium channel blockers have been demonstrated to have pleiotropic effects leading to the improvement of endothelial function and to the reduction of central aortic pressures (56). These effects are not directly linked to the antagonism of voltage-dependent calcium channels but rather are associated to the reduction of ET-1, monocyte chemoattractant protein-1 and C-reactive protein (57). Third generation beta-blockers with α1-adrenergic receptor antagonist activity have also been shown to improve endothelial function through antioxidant mechanisms and cause NO-dependent vasodilation (57) (Figure 2). Furthermore, endothelin receptor antagonists, might represent feasible future therapeutic agents to prevent endothelial dysfunction, vascular remodeling and organ damage in hypertension (58).

## Relationship Between Endothelium and Extracellular Vesicles

Extracellular vesicles (EVs) are released in plasma from cells after the fusion of multivesicular bodies with the plasmic membrane and can deliver their cargo, including mRNA, microRNA (miRNA), small amounts of DNA, transcription factors, cytokines, and growth factors, to other cells in remote locations (59, 60). EVs can also be released into the extracellular space by neighboring cells through paracrine mechanisms along with the systemic release in plasma (61). Available findings have shown a correlation between endothelial dysfunction and circulating levels of EVs, particularly in patients with hypertension, coronary artery disease (CAD) and diabetes, although conflicting evidence exists with respect to their protective or harmful role (62–64). EVs have been identified as potential novel biomarkers and bioactivators in the development of hypertension affecting vascular tone in patients with endothelial dysfunction (65). It has been shown that EVs may reduce endothelial-dependent vasodilation and impair acetylcholine (ACh) induced vasorelaxation in a concentration-dependent manner. However, it has not been completely clarified how circulating EVs may affect resistance artery function during the basal state and when overt hypertension may occur (66). A recent study has demonstrated that an enriched EVs preparation from normotensive individuals (humans or rats) impair vasodilation in response to endothelial-dependent vasodilators, potentially through L-NAME inhibitory effects on eNOS. These findings support a paracrine/endocrine role of circulating EVs in the regulation of vascular tone in resistance arteries (67). Other animal studies showed that the dilatation of mouse mesenteric arteries induced by shear stress was impaired by the infusion of EVs isolated from diabetic patients (68) and that endothelial derived EVs decreased NO and increased ROS production, impairing ACh-mediated vasorelaxation, at aortic ring level (69).

On the other hand, EVs have shown beneficial effects on endothelial cells by inhibiting hyperproliferative pathways, through the activation of eNOS signaling mediated by miRNA (70, 71). Moreover, MiR-143/miR-145 contained in EVs has been shown to reduce atherosclerotic lesion formation in the aorta of ApoE–/– mice (72). MiR-19a72 and miR-23b70 mediate the atheroprotective laminar shear stress-induced cell cycle arrest via a decrease in E2F1 and hypophosphorylation of retinoblastoma or directly targeting cyclin D1 (73).

Interestingly, an increasing body of evidence have shown that long non-coding RNA (lncRNAs) can be selectively packaged into EVs and may act as regulators of endothelial function which may represent a promising therapeutic tool, although further studies are required to clarify the specific targets (74, 75).

## Potential Future Therapeutic Strategies Based on Nanoparticles

Over the past decades several non-pharmacological (i.e., diet, antioxidants, and vitamin supplementation) and pharmacological approaches have been suggested in order to improve endothelial function as mentioned previously. Recently, selective, and more specific approaches such as nanoparticles have been proposed. Nanomedicine is emerging as an innovative approach with the aim to target specific endocytic pathways throughout the formulation of different nanoparticles, encapsulating therapeutic agents with enhanced bioavailability and ensuring treatment effectiveness. Indeed, the challenge to drug delivery in the endothelium consists in the selection of appropriate targets and in the design of nanoparticle-formulations with appropriate binding-properties to the vascular endothelium in micro, small, medium and in large vessels against continuous flowing blood (76, 77). In particular, the delivery through nanoparticles of many clinically used drugs such as antihypertensive agents, statins, antidiabetic drugs and interleukin 1β monoclonal antibodies may represent a potential target for treatment of endothelial dysfunction thus yielding potential new therapeutic approaches (78).

Future application may include the use of several types of small molecules that target complementary epigenetic pathways. More specifically, histone deacetylase inhibitors, DNA methyltransferase inhibitors, histone methyltransferases and demethylase inhibitors have been demonstrated to play an essential role in the regulation of endothelial stem/progenitor cell functions through modifying chromatin structure. In such a context, nanoparticles might be used to modulate the activities of epigenetic enzymes to enhance the vascular repair function of endothelial cells (79).

## Conclusions

Several lines of evidence support the role of endothelium in physiology of peripheral arteries. Impairment of vascular flow, RAS activation, oxidative stress and inflammation have been demonstrated to play a fundamental role in the development of endothelial dysfunction in hypertensive patients, leading to vascular remodeling, atherosclerotic plaques progression and eventually increased risk of CV events (Figure 1).

The modulation of vascular inflammation through RAS blockers and other antihypertensive drugs is a well assessed therapeutic approach to improve vascular function and remodeling.

Recent evidence suggests that EVs have attracted increasing interest both as biomarkers or mediators of disease, as well as vehicles for delivering bioactive molecules, such as miRNA and drugs interacting with RAS, with potential beneficial effects on the endothelium. Hence EVs have emerged as important regulators of endothelial function and potentially as a promising novel therapeutic approach to improve endothelial dysfunction.

Moreover, a better molecular understanding of organ vasculature-bed heterogeneity and of organ/tissue microenvironment-governed endothelial cell phenotypic changes may represent the lead foundation for innovative tissue specific therapies (80).

## Author Contributions

GG, MV, and CS substantially contributed to the conception and design, drafted the article, and approved the final version to be published.

## Conflict of Interest

The authors declare that the research was conducted in the absence of any commercial or financial relationships that could be construed as a potential conflict of interest.

## Publisher's Note

All claims expressed in this article are solely those of the authors and do not necessarily represent those of their affiliated organizations, or those of the publisher, the editors and the reviewers. Any product that may be evaluated in this article, or claim that may be made by its manufacturer, is not guaranteed or endorsed by the publisher.
